# Psoriasis and Cardiovascular Diseases: A Literature Review to Determine the Causal Relationship

**DOI:** 10.7759/cureus.2195

**Published:** 2018-02-15

**Authors:** Shanu Jindal, Nitin Jindal

**Affiliations:** 1 Public Health Education, University of North Carolina-Greensboro; 2 James K. Elrod Department of Health Administration, Louisiana State University, Shreveport, LA.

**Keywords:** cardiovascular diseases, psoriasis

## Abstract

Psoriasis is a chronic, complex autoimmune disease characterized by erythematous, scaly patches over extensor aspects of skin and is associated with joint involvement in about one-third of patients. An association between psoriasis and cardiovascular diseases (CVD) has been a topic of dilemma, and many studies have shown an increased risk of cardiovascular morbidity in patients with psoriasis. There is increasing evidence that psoriasis is associated with higher risk of CVD and increased prevalence of cardiovascular risk factors, as compared with the general population. We provide an extensive review of the literature and adhere to Gordis guidelines to show a positive association between psoriasis and cardiovascular events.

## Introduction and background

Cardiovascular diseases (CVD) refer to a group of diseases involving the heart and blood vessels. Heart disease is the number one silent killer in the world for both men and women. Stroke ranks second globally and is a leading cause of severe disability. Each year, CVD kills more than 786,000 Americans, which is larger than the population of several states (Alaska, North Dakota, Vermont, and Wyoming) [1]. About 610,000 Americans die from heart disease each year, which is about one in every four deaths [2].

Coronary heart disease (CHD) is the most common type of heart disease, killing more than 370,000 people annually. In the United States, someone has a heart attack every 43 seconds [2]. Each minute, someone in the United States dies from a heart disease-related event. It is also the leading cause of death for people of most racial/ethnic groups in the United States, including African Americans, Hispanics, and whites. For Asian Americans or Pacific Islanders and American Indians or Alaska Natives, heart disease is second only to cancer [2]. CHD alone costs the United States $108.9 billion each year.

In recent years, the dominance of chronic diseases as significant contributors to total global mortality has emerged. By 2005, the total number of CVD deaths, mainly coronary heart disease, stroke, and rheumatic heart disease, had increased globally to 17.5 million from 14.4 million in 1990. Of these, 7.6 million were attributed to CHD and 5.7 million to stroke [3]. A recent review of the global burden of high blood pressure found that approximately 54% of stroke, 47% of ischaemic heart disease, and 25% of other CVDs were attributable to hypertension. This equates to an annual burden of approximately 7.6 million deaths, or 13.5% of the total number of annual global deaths, attributable to high blood pressure [4]. By 2030, researchers project that non-communicable diseases will account for more than three-quarters of deaths worldwide; CVD alone will be responsible for more deaths in low-income countries than infectious diseases, maternal and perinatal conditions, and nutritional disorders combined [3]. In 2006, data from the American Heart Association (AHA) revealed that the prevalence of CVD for both sexes was 36.3%. It was estimated that 80 million American adults (1 out of 3) had a form of CVD. In 2006, the prevalence of CVD in females (34.9%) was lower than in males (37.6%). The prevalence of CVD in black males (45.9%) and black females (45.9%) was higher than white males (37.8%) and females (33.3%). CHD is the most prevalent form of CVD (7.6%), affecting 16.8 million people in 2006 [5]. High blood pressure, high low-density lipoprotein (LDL) cholesterol, and smoking are key heart disease risk factors for heart disease. About half of Americans (49%) have at least one of these three risk factors [2]. AHA states that CVD and stroke are largely preventable by lowering the risks. This can be done by adhering to what we call “Life’s Simple 7”: not smoking, being physically active, and maintaining a healthy body weight, eating a healthy diet, and controlling blood pressure, cholesterol and blood sugar [1]. Examination of CHD mortality trends across countries reveals considerable variability in the shape and magnitude of CHD epidemics since the 1950s. From 1996 to 2005, age-standardized incidence of CHD decreased by the average percentage change of 2.2% in men and 2.3% in women per year. Age-standardized all-cause mortality among those with CHD decreased by 4.5% in men and 3.4% in women per year showing an increase in preventive and treatment methods, while age-standardized prevalence increased by 1.3% in men and 1.7% in women per year demonstrating the need for early preventive measures and study of other associated factors as top priority [6]. Recent studies suggest that psoriasis is a systemic disorder associated with CVD risk factors, such as obesity, hypertension, and dyslipidemia [7].

CVD is likely caused by atherosclerosis—a chronic inflammatory disease of blood vessels. Innate as well as adaptive immune responses have been identified during the course of atherosclerosis, involving the Th-1 and Th-17 pathways [8]. Similarly, psoriasis is also a chronic systemic inflammatory disease characterized by topical skin lesions. It demonstrates the implication of Th-1, Th-17, and Th-22 mediated inflammation in inducing deregulated production of cytokines, like lL-17, interferon- and tumor necrosis factor. This plausible biological explanation suggests a link between psoriasis and CVD through common immunological and inflammatory pathways [9].

Psoriasis is one of the most common immune-mediated inflammatory disorders, with prevalence estimates worldwide ranging from 0.91% (United States) to 8.5% (Norway) [10]. About seven million Americans are plagued by this itching and scaling, and many of them have serious complications involving other organs. The extent varies from a few scaly plaques at extensor sites to whole-body involvement; it does not start in the skin, and its damage may be more than skin deep. In about a third of cases, psoriasis runs in families, and at least nine genetic abnormalities have already been associated with psoriasis. It is also linked to environmental factors such as psychological stress, obesity, smoking, alcohol, strep throat, viral infections, lack of sunlight, and even certain medications (anti-malaria drugs, lithium, beta blockers, and others) [11].

## Review

Psoriasis is a disease that is characterized by topical skin lesions as well as an increased risk for CVD. There is increasing evidence that patients with psoriasis are more associated with various CVD risk factors (hypertension, diabetes, obesity, and dyslipidemia) and have a higher risk of non-cardiac vascular diseases (carotid, peripheral artery, and chronic kidney diseases), as compared with the general population. The associations are even higher in patients with severe psoriasis and those with psoriatic arthritis [12]. The possible relationships between cardiovascular risk factors and psoriasis have been investigated in numerous articles over the past 100 years and results have been variable; however, recent comprehensive meta-analysis, cohort studies, etc. using categorical outcomes have confirmed that the association is statistically significant [13]. But still, more long-term prospective studies are needed to evaluate the association. Based on Gordis guidelines for judging whether an association is causal, this paper will provide a review of the relevant literature regarding the potential association between psoriasis and CVD. Nine key points in the Gordis guidelines are: a temporal relationship, a strength of the association, a dose-response relationship, replication of study findings, biologic plausibility, consideration of alternate explanations, cessation of exposure, consistency with other knowledge, and specificity of the association [14].

Temporal relationship

Psoriatic arthritis (PsA) occurs almost exclusively in the context of psoriasis. In two cohort studies of psoriatic arthritis, mortality was increased, and CVD was the leading cause of death. Patients with PsA were more likely to develop hypertension, myocardial infarction, angina, and were at greater risk for cardiovascular morbidity as compared with general population. From a cardiologist's perspective, risk factors for CVD are often present in patients with psoriasis, including elevated LDL-cholesterol with low HDL-cholesterol, hypertension, elevated highly sensitive-C-reactive protein (hs-CRP), diabetes, obesity, metabolic syndrome, cigarette smoking, and a history of coronary artery disease [15]. PsA may also be an independent risk factor for CVD. Endothelial dysfunction and increased vascular intima/media thickness have been observed in patients with PsA without cardiovascular risk factors or clinically evident CVD. It is well established that severe psoriasis is associated with an increased prevalence of traditional risk factors for CVD and is also an independent risk factor for myocardial infarction, stroke, and cardiovascular mortality [16, 17]. A cross-sectional study was held in a Korean population to determine the association between psoriasis and CVD. A higher prevalence of metabolic syndrome (p=0.021) and CVDs (p=0.044) was observed among the patients with psoriasis as compared to that of the controls. To evaluate the absolute risk of major coronary events, the Framingham 10-year risk score was determined for the patients with psoriasis. The U.S. Preventive Services Task Force proposed that aspirin should be used to prevent CHDs when the five-year risk score attained from the Framingham Heart Study is 3% or greater, or the 10-year risk score is 6% or greater, after consultation with the patients. In this study, a substantial portion of patients with psoriasis met the criteria for using aspirin, since 73 patients (37.0%) with psoriasis exhibited a 6% or greater Framingham 10-year risk [18].

Strength of association

A report utilizing the General Practice Research Database (GPRD) found that risk factors for CVD occurred more frequently in psoriasis patients compared with the general population, including incident diabetes, hypertension, obesity, hyperlipidaemia, myocardial infarction (MI), angina, atherosclerosis, and peripheral vascular disease [19]. This association has been confirmed in a recent United States (US) study that found an increased risk for ischemic heart disease in psoriasis patients {odds ratio (OR), 1.78; 95% confidence interval (CI), 1.51–2.11}, cerebrovascular {OR, 1.70; 95% CI 1.33–2.17}, and peripheral vascular events {OR, 1.98; 95% CI 1.32–2.82} [20]. In a population-based, cross-sectional study in the United Kingdom (UK), psoriasis was associated with metabolic syndrome, {adjusted OR 1.41, 95% confidence interval (CI) 1.31-1.51}, varying in a 'dose-response' manner, from mild {adjusted OR 1.22, 95% CI 1.11-1.35} to severe psoriasis {adjusted OR 1.98, 95% CI 1.62-2.43} [21].

Dose-response relationship

Heart failure (HF) is independently associated with several cardiovascular risk factors and is a major cause of cardiovascular morbidity and mortality. A nationwide cohort study of psoriasis patients was compared with the background population. The study included the entire Danish population aged ≥18 years followed from January 1, 1997 until HF, death, or December 31, 2011. In the study period, 66389 patients with new-onset psoriasis, including 11242 patients with severe psoriasis, were identified. The overall incidence rates of new-onset HF were 2.82, 4.22, and 4.70 per 1000 person-years for the reference population, mild psoriasis, and severe psoriasis, respectively. This concludes that psoriasis was associated with a disease severity-dependent increased risk of new-onset HF [22]. Psoriasis is associated with the metabolic syndrome in a ‘‘dose-response manner”, with a 22% increase in the odds of developing the metabolic syndrome in those with mild psoriasis, 56% increase in those with moderate disease, and a 98% increase in those with severe psoriasis [21].

Replication of findings

Through research we have found that there is consistency in different studies and populations that show a relationship between psoriasis and CVD. Psoriasis affects approximately 1-2% of the population worldwide (a collective 12 million individuals within the United States of America (USA) and Europe) [23]. The studies stated above in the temporal relationship, the strength of association, and dose-response relationship demonstrate the broad range of study population and the causal relationship between psoriasis and CVD. Recent studies have examined the risk of cardiovascular events in patients with psoriasis according to the Framingham cardiovascular risk prediction score. These studies documented that a high proportion of patients with psoriasis were at substantially increased risk and are potential candidates for pharmacological cardiovascular primary prevention [20]. The Framingham risk score is a tool to predict the absolute risk of major coronary and cerebrovascular events at five and 10 years in adults from 30 to 74 years of age by stratifying patients into three risk categories: patients scoring less than 10% are at low risk, those between 10% and 20% have a moderate risk, and those scoring 20% or more are at high risk. This score is appropriate for United States, Australia, and New Zealand populations [24]. In a hospital-based study in Germany, individuals with psoriasis had elevated rates of diabetes, hyperlipidemia, coronary artery disease, and arterial hypertension compared with controls [25]. An Italian study likewise showed that metabolic syndrome, hypertriglyceridemia, and abdominal obesity were more common in psoriasis patients than in controls [26].

Biologic plausibility

Metabolic syndrome is a chronic inflammatory state associated with markedly increased cardiovascular mortality. Severe psoriasis is associated with an increased prevalence of metabolic syndrome, an emerging but variable health issue estimated to affect 25% of the population of the United States [23]. The systemic inflammation present in a psoriasis patient has been shown to yield hyperhomocysteinemia, elevated highly sensitive-C-reactive protein (hs-CRP), platelet hyperactivity, and elevated blood inflammatory cytokines (e.g., IL-6, TNF-a, IL- 23, IL-17, IL-20, IL-22), all of which may also contribute to the increased incidence of ischemic heart disease [27]. Inflammatory markers such as Th-1 cytokines (intracellular adhesion molecule-1, TNF-α) play a role not only in the pathogenesis of psoriasis, but also in the pathogenesis of metabolic syndrome, obesity, atherosclerosis, and myocardial infarction. Patients with psoriasis have elevated levels of C-reactive protein (CRP), which has been independently associated with an increased risk of CVD [15].

Consideration of alternate explanations

Psoriasis is associated with other disease states and behaviors that potentially increase morbidity and mortality, and lower the quality of life. Evidence continues to accumulate to support the association of psoriasis with established co-morbidities that increase the risk of CVD, including components of metabolic syndrome such as hypertension, diabetes, dyslipidemia, and obesity [28]. Some population-based cohort studies also suggest that individuals with psoriasis, in common with other inflammatory autoimmune diseases such as rheumatoid arthritis and inflammatory bowel disease, are at an increased risk of CVD [23]. These findings have led some investigators to hypothesize, first, that the documented association between psoriasis and CVD reflects a causal relationship (i.e., systemic inflammation is driving CVD risk), and, second, that the use of systemic treatment for psoriasis with methotrexate and biological drugs will, therefore, reduce CVD risk demonstrating the cessation of exposure [29].

Cessation of exposure

It is well known that increased cholesterol levels are associated with cardiovascular risk, and that lowering cholesterol can decrease this risk. For this reason, the Adult Treatment Panel III (ATP III) guideline, recommends that the cholesterol levels should be regulated by focusing on LDL cholesterol. The cross-sectional study in the Korean population mentioned before included 197 patients with psoriasis and 401 controls. The Framingham 10-year risk score was calculated for the 159 patients (80.7%) with psoriasis and who were aged 20 and older and who do not have heart disease or diabetes. The study found a higher prevalence of metabolic syndrome (17.8%, p=0.021), CVD (4.6%, p=0.044), hypertension (32.5%, p=0.000), and hyperlipidemia (22.3%, p=0.025) in patients with psoriasis, as compared with that of the controls. Seventy-three (37%) patients with psoriasis had LDL levels higher than the target LDL level proposed by the ATP III guideline. To maintain proper LDL levels, 25.3% of the psoriasis patients needed lifestyle changes and 11.7% needed drug therapy [18].

## Conclusions

Based on the previously mentioned studies and findings, it is well established that psoriasis is associated with increased risk of CVD and increased prevalence of cardiovascular risk factors. More studies are needed to evaluate the true causal relationship between psoriasis and CVD. Formal, long-term prospective studies are required in the psoriatic population to identify those patients at the greatest risk for comorbidities and to better understand the true association with the full spectrum of psoriasis. Screening practices and treatment aimed at cardiovascular risk factors and disease remain a challenge in clinical practice. It is necessary to enhance the awareness of dermatologists, cardiologists, and general practitioners on sub-clinical atherosclerosis in psoriasis patients, so that general preventive measures and early therapeutic interventions can be implemented, reducing the burden of high mortality events such as acute myocardial infarction and stroke. Specific needs for follow-up are related to the natural history of psoriasis and the chronology of the association with the comorbidities.
